# PIK3CA amplification and PTEN loss in diffused large B-cell lymphoma

**DOI:** 10.18632/oncotarget.19889

**Published:** 2017-08-03

**Authors:** Wenli Cui, Mingfu Ma, Shutao Zheng, Zhiping Ma, Liping Su, Wei Zhang

**Affiliations:** ^1^ Department of Pathology, First Affiliated Hospital of Xinjiang Medical University, Urumqi 830011, Xinjiang, PR.China; ^2^ Clinical Medical Research Institute, First Affiliated Hospital of Xinjiang Medical University, Urumqi 830011, Xinjiang, PR.China; ^3^ State Key Lab Incubation Base of Xinjiang Major Diseases Research, First Affiliated Hospital of Xinjiang Medical University, Urumqi 830011, Xinjiang, PR.China

**Keywords:** diffuse large B-cell lymphoma (DLBCL), PTEN, PIK3CA, FISH, prognosis

## Abstract

Although it has been known that PIK3CA was amplified and PTEN was deficient on protein level in DLBCL, the clinicopathological significance of PIK3CA and PTEN genetic change on DNA level hasn’t been established. Here, in our present study, to understand the clinical significance of genetic status of PIK3CA and PTEN in DLBCL, fluorescent in-situ hybridization (FISH) was employed to evaluate the genetic change of PIK3CA and PTEN in clinical sample tissues consist of 205 cases. Incidentally, to understand the clinicopathological significance of genetic change of PIK3CA and PTEN, Cross-table analysis was used to analyze the association between genetic change of PIK3CA and PTEN versus clinicopathological variables available to us, including age, gender, size, location, international prognosis index, performance state, B-symptom, clinical stage, Extra nodal site, concentration of lactate dehydrogenase, therapeutic effects, treatment and overall prognosis. It was found that PIK3CA was amplified and PTEN was deficient on DNA level, the percentage of amplification and loss was 12.7% (26/205) and 12.2% (25/205), respectively. Additionally, no significant association was observed between genetic change of PIK3CA and PTEN versus clinicopathological variables available. Nor was the significant correlation found between loss of PTEN versus PIK3CA amplification. Our results suggest that PTEN deficiency and amplification of PIK3CA on DNA level was an event in the pathogenesis of DLBCL.

## INTRODUCTION

Diffuse large B cell lymphoma (DLBCL) is one of the most common types of aggressive B cell non-Hodgkin lymphomas [1, 2]. DLBCL is highly prevalent and is a heterogeneous disease that can be subdivided into two main lymphoma categories, that is germinal center B cell-like subtype (GCG) and activated B cell-like subtype (ABC), which differs in the gene expression profiles and clinical outcomes [3]. Despite treatment of DLBCL with standard R-CHOP that appears to be superior to CHOP chemotherapy that can cure some patients [1, 4], there are still, approximately 30%-40% of patients were shown to be going to develop relapsed disease that remains the major cause of the poor survival. Therefore, the important issue is to understand the underlying mechanism why refractory DLBCL occurs to patients [5].

Studies showed that constitutive activation of the PI3K/AKT signaling pathway [6, 7] as well as its inhibitor, phosphatase and tensin homolog (PTEN) [8], in GCB-DLBCL plays a central role in promoting survival and chemotherapy-resistance and represents a rational therapeutic target in relapsed or refractory GCB-DLBCL. Deregulation of the PI3K/AKT pathway by the inactivation of PTEN, was found in 55 % of GCB-DLBCL cases, but only in 14 % of non-GCB-DLBCL and worsens prognosis [8]. Thus, the both of PTEN and PI3K/AKT signaling pathway has emerged as promising therapeutic targets for relapsed DLBCL [9]. Some inhibitors [7, 8] of PI3K subunit has been currently evaluated in an ongoing early phase I study as a single-agent in patients with relapsed/refractory DLBCL.

Gene amplification of PIK3CA has been considered to contribute to the pathogenesis of DLBCL and mantle cell lymphoma [10, 11]. In our previous study, we’ve evaluated the amplification status of both catalytic and regulatory subunits of PI3K/AKT signaling pathway on DNA level using NanoString nCounter platform on a basis of 60 cases of clinical samples of DLBCL, finding that copy number variation of PIK3CA subunit was amplified [12]. Nevertheless, as for the copy number variation of PTEN gene in DLBCL on DNA level, it remains unknown that hasn’t been established on account of scarce report [13] available regarding PTEN in DLBCL. Given this, we’ve determined to extend the detection of copy number variation of PTEN as well as PIK3CA on DNA level in larger sample tissues of DLBCL and tried to analyze the clinicopathological significance of genetic variation PTEN and PIK3CA.

## RESULTS

### PIK3CA was found to be amplified in DLBCL

**Figure 1:**
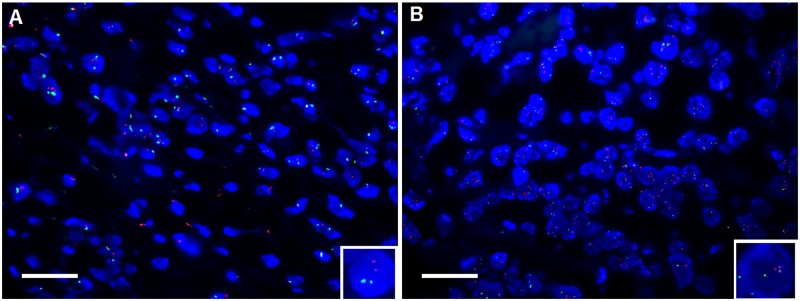
Shown was the amplification of PIK3CA in DLBCL tissue using fluorescent in situ hybridization (FISH) **(A)** non-amplification of PIK3CA. **(B)** amplification of PIK3CA. Red spectrum stands for the probe of PIK3CA whereas Green represents the probe of centromere at the same chromosome as PIK3CA does, used as internal control; blue denotes the nucleus staining with DAPI. In the nucleus where the ratio of red signals to green signals equals to one (inset) that was present that was defined as normal or non-amplification status whereas the ratio of red signals to green signals was more than one (inset) was defined as amplification. The magnification was 400 fold; scale bar was 100μm. here shown was the representative figure selected from candidates.

To understand the genetic alteration of PIK3CA gene in DLBCL, fluorescent in-situ hybridization (FISH) was performed. In our setting, only two green and two red fluorescent signals that can be observed in the nucleus of DLBCL cells that was defined as normal or non-amplified status of PIK3CA, for those whose ratio of red signals to green signal was more than one were defined as amplified PIK3CA (Figure 1). According to the cut-off value we’ve set, among 205 cases we’ve enrolled, only 26 cases whose PIK3CA gene status was found to be amplified. The amplification percentage was 12.7% (26/205), while the reminder was non-amplified whose percentage was 87.3% (179/205).

### PTEN was shown to be deficiency in DLBCL

**Figure 2:**
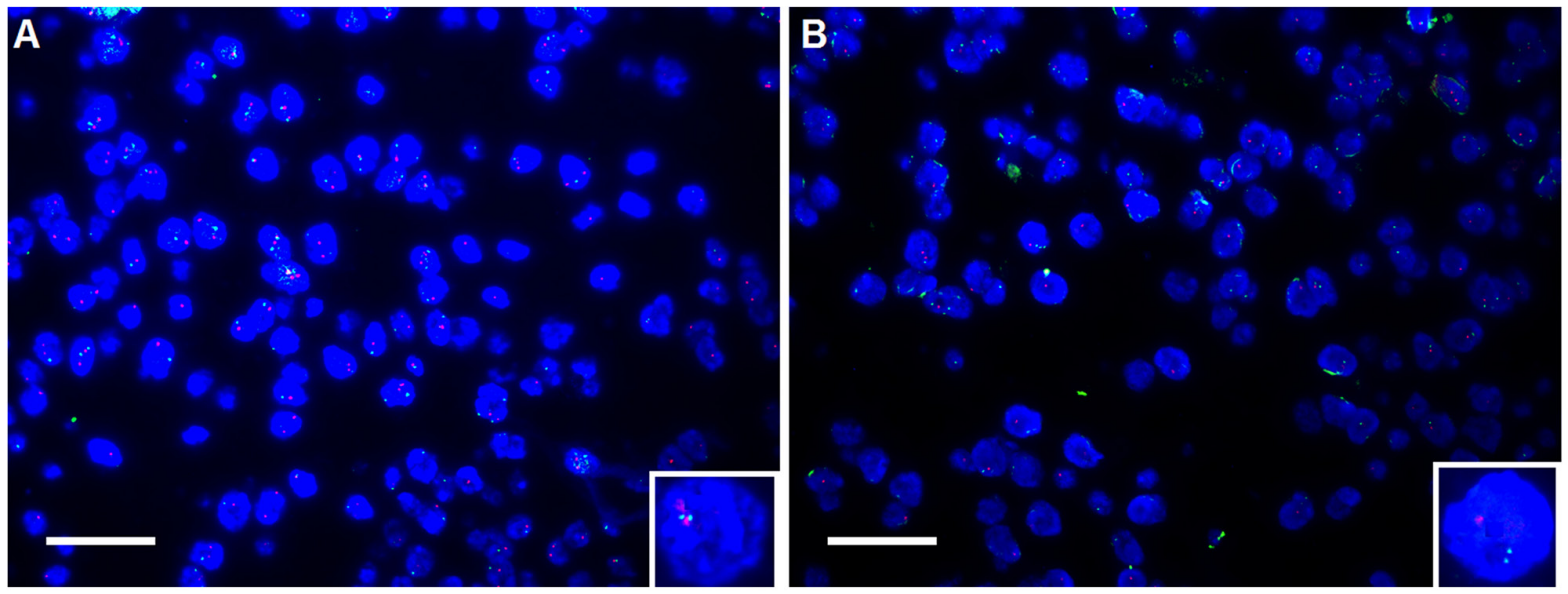
Shown was the deficiency of PTEN in DLBCL tissue using fluorescent in situ hybridization (FISH) **(A)** non-deficiency of PTEN. **(B)** deficiency of PTEN. Similarly, red spectrum stands for the probe of PTEN, and Green represents the probe of centromere at the same chromosome as PTEN does; blue represents the nucleus staining with DAPI. In the nucleus where the ratio of red signals to green signals equals to one (inset) that was observed that was defined as non-deficiency status or normal whereas the ratio was less than one (inset) was defined as deficiency. The magnification was 400 fold; scale bar was 100μm. here shown was the representative figure selected from candidates.

Likewise, to understand the genetic change of PTEN in DLBCL, FISH detection was extended from PIK3CA to PTEN. That is, FISH test for PTEN was carried out in the same sample of DLBCL as PIK3CA did. In this setting, the cut-off value we’ve set was the ratio of red signal (which stands for PTEN) to green signal (centromere probe, used as internal control) less than 1 was defined as PTEN deficiency (Figure 2). Following the cut-off value we’ve set, of 205 cases, PTEN loss occurred to 25 cases whose deficient percentage was 12.2% (25/205).

### PIK3CA amplification and PTEN deficiency may be independent of overall prognosis and clinicopathological variables available in our setting

**Figure 3:**
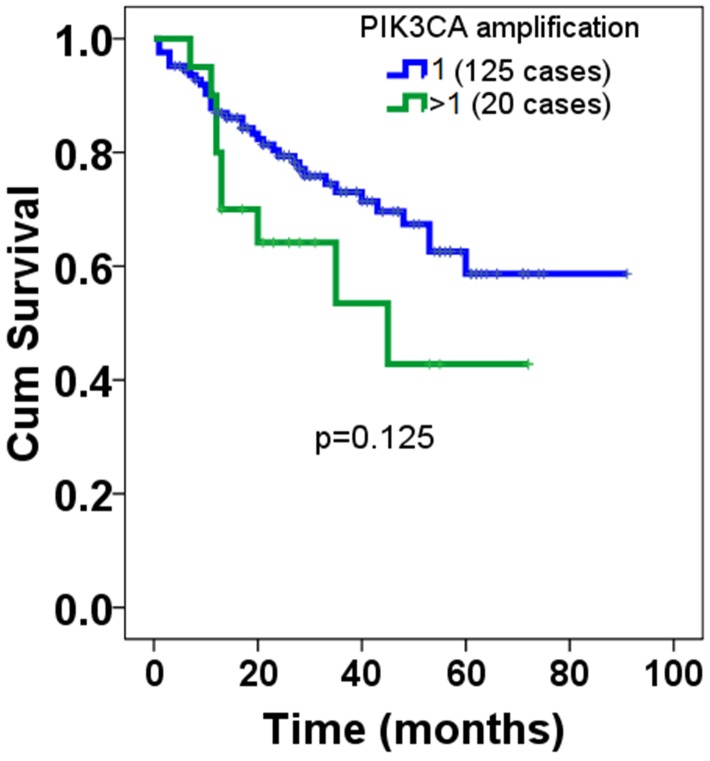
Overall prognosis of PIK3CA amplification in patients with DLBCL Among the DLBCL samples we’ve enrolled, totaling 205, only 145 cases whose overall prognosis information were available. Of 145 cases, 20 were found to be PIK3CA amplification, whereas the remainder was non-amplification. The cut-off value was set at one that the ratio of red signals to green signals; those whose ratio was more than one was defined as PIK3CA amplification, while those whose ratios equal to one were non-amplification. Log-Rank test was used to analyze the statistical difference of overall prognosis between patients with PIK3CA amplification and those with non-amplification.

**Figure 4:**
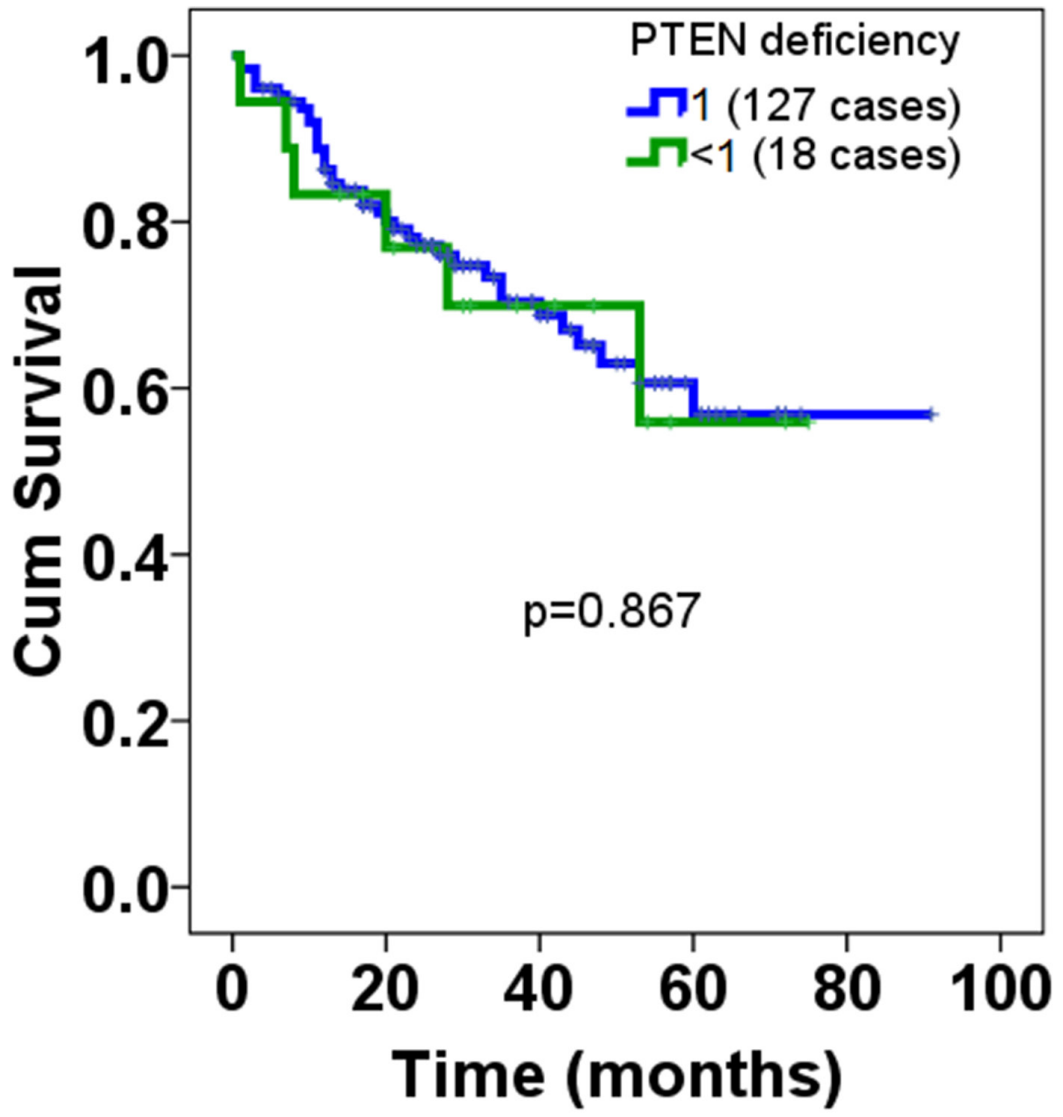
Overall prognosis of PTEN deficiency in patients with DLBCL Among the DLBCL samples we’ve enrolled, totaling 205, only 145 cases whose overall prognosis information were available. Of 145 cases, 18 were found to be PTEN deficiency, the rest was non-deficiency. The cut-off value was set at one that the ratio of red signals to green signals; as long as those whose ratios were less than one it was defined as deficiency, while those whose ratios equal to one were defined as non-deficiency. Log-Rank test was used to analyze the statistical difference of overall prognosis between patients with PTEN deficiency and those with loss.

Next, to understand the clinicopathological significance of PIK3CA amplification and PTEN deficiency, we’ve analyzed the correlation using Cross-Table statistical approach between genetic alterations of PIK3CA and PTEN versus clinicopathological variables available to us, including age, size, international prognosis index (IPI), performance state (PS), B-symptom, clinical stage and so forth. It was found that there was no significant correlation between PIK3CA amplification and clinicopathological variables (Table 1) available to us, including age (p=0.933), gender (p=0.840), size (p=0.440), location (p=0.117), subtype (p=0.659), international prognosis index (IPI) (p=0.956), performance state (PS), B-symptom (p=0.682), Clinical stage (p=0.298), Extra nodal site (p=0.173), concentration of lactate dehydrogenase (LDH) (p=0.684), therapeutic effects (p=0.552) and treatment (p=0.984), Nor was significant correlation observed for PTEN deficiency (Table 2). Considering the regulation that has been established between PTEN and PI3K signaling pathway, subsequently we’ve tried to analyze the correlation using Spearman Correlation analysis method between amplification of PIK3CA and PTEN deficiency that were detected in the same cohort. It was observed that there was no significant correlation between amplification of PIK3CA and PTEN deficiency but, notably there was a trend towards the significance (p=0.087). Having understood the clinicopathological significance of PIK3CA amplification and PTEN deficiency, we’ve subsequently explored the prognostic significance of PIK3CA amplification and PTEN deficiency. It was shown that there was no markedly difference of overall prognosis between DLBCL patients with PIK3CA amplification and non-amplification (Figure 3). No significant difference of overall prognosis was observed between DLBCL patients with PTEN deficiency and non-deficiency, either (Figure 4). Despite no significant difference of prognosis was found between PIK3CA amplification and non-amplification, there was the trend existed that patients with PIK3CA amplification seem to have had shorter survival relative to those without PIK3CA amplification. As for PTEN deficiency, even there was no trend at all.

**Table 1:** The correlation between PIK3CA amplification and clinicopathological variables

	Total	PIK3CA amplification (%)	*P*
Age			
≤60	124	16 (12.9)	0.933
>60	80	10 (12.5)	
Gender			
male	122	15 (12.3)	0.840
female	83	11 (13.3)	
Size (diameter, cm)			
≤10	168	17 (10.1)	0.440
>10	22	4 (18.2)	
Location			
extra nodular	138	14 (10.1)	0.117
Intra nodular	67	12 (17.9)	
Subtype			
GCB	110	15 (13.6)	0.659
Non-GCB	95	11 (11.6)	
IPI			
0-1	106	12 (11.3)	0.956
2	44	6 (13.6)	
3	22	2 (9.1)	
4-5	17	2 (11.8)	
PS			
<2	168	22 (13.1)	0.398
>2	33	2 (6.1)	
B-symptom			
presence	100	11 (11.0)	0.682
absence	101	13 (12.9)	
Clinical stage			
I/II	115	11 (9.6)	0.298
III/IV	76	11 (14.5)	
Extra nodal site			
≤2	162	16 (9.9)	0.173
>2	29	6 (20.7)	
LDH (U/L)			
≤240	104	13 (12.5)	0.684
>240	85	9 (10.6)	
Therapeutic effect			
CR+PR	92	11 (12.0)	0.552
PD+SD	20	4 (20.0)	
Treatment			
CHOP	98	12 (12.2)	0.984
R-CHOP	81	10 (12.3)	
PTEN deficiency			
No	180	26 (14.4)	0.087
Yes	25	0 (0)	

GCB stands for germinal center B cell; IPI represents international prognosis index; PS denotes performance status; LDH means lactate dehydrogenase; in therapeutic effect, CR means complete response and PR denotes partial response; while PD and SD which were abbreviated for progressive disease and stable disease, respectively. Among 205 cases of samples we’ve enrolled, there was 1 case whose age was unavailable; 15 cases whose size information was unavailable; 16 cases whose IPI was unavailable; 4 cases whose PS was unavailable; 4 cases whose B-system was unavailable; 14 cases whose clinical stage and Exln2 were unavailable; 16 cases whose LDH level was unavailable; 93 cases whose therapeutic effect was unavailable; 26 cases whose treatment was unavailable.

**Table 2:** The correlation between PTEN deficiency and clinicopathological variables

	Total	PTEN deficiency (%)	*P*
Age			
≤60	124	14 (11.3)	0.793
>60	80	10 (12.5)	
Gender			
male	122	12 (9.8)	0.211
female	83	13 (15.7)	
Size (diameter, cm)			
≤10	168	21(12.5)	0.645
>10	22	2 (9.1)	
Location			
Extra nodular	138	20 (14.5)	0.149
Intra nodular	67	5 (7.5)	
Type			
GCB	110	16 (14.5)	0.268
Non-GCB	95	9 (9.5)	
IPI			
0-1	106	14 (13.2)	0.065
2	44	3 (6.8)	
3	22	1 (4.5)	
4	17	5 (29.4)	
PS			
<2	168	18 (10.7)	0.226
>2	33	6 (18.2)	
B-symptom			
presence	101	11 (10.9)	0.645
absence	100	13 (13)	
Clinical stage			
I/II	115	15 (13.0)	0.601
III/IV	76	8 (10.5)	
Extra nodal site			
≤2	162	18 (11.1)	0.350
>2	29	5 (17.2)	
LDH (U/L)			
≤240	104	15 (14.4)	0.294
>240	85	8 (9.4)	
Therapeutic effects			
CR+PR	92	14 (15.2)	0.546
PD+SD	20	2 (10.0)	
Treatment			
CHOP	98	12 (12.2)	0.984
R-CHOP	81	10 (12.3)	
PIK3CA amplification			
No	178	25 (14.0)	0.087
Yes	26	0 (0)	

GCB stands for germinal center B cell; IPI represents international prognosis index; PS denotes performance status; LDH means lactate dehydrogenase; in therapeutic effect, CR means complete response and PR denotes partial response; while PD and SD which were abbreviated for progressive disease and stable disease, respectively. Among 205 cases of samples we’ve enrolled, there was 1 case whose age was unavailable; 15 cases whose size information was unavailable; 16 cases whose IPI was unavailable; 4 cases whose PS was unavailable; 4 cases whose B-system was unavailable; 14 cases whose clinical stage and Exln2 were unavailable; 16 cases whose LDH level was unavailable; 93 cases whose therapeutic effect was unavailable; 26 cases whose treatment was unavailable.

## DISCUSSION

Although previous studies have reported that the genetic changes of PIK3CA and PTEN occurred in DLBCL, little has been known about the clinicopathological significance of genetic changes of both PIK3CA and PTEN on DNA level in DLBCL. In the present investigation, both amplification of PIK3CA and PTEN loss were shown to have occurred on DNA level with the help of FISH approach in DLBCL. The percentage of amplification of PIK3CA and loss of PTEN was 12.7% (26/205) and 12.2% (25/205), respectively. Moreover, no significant association was observed between copy number variation of PIK3CA and PTEN gene versus clinicopathological variables available to us. Nor was association found with overall prognosis. There was the trend towards statistical significance (p=0.087) in spite of no significant correlation was observed between amplification of PIK3CA versus loss of PTEN on DNA level. Our results suggested that both amplification of PIK3CA and loss of PTEN were events in the pathogenesis of DLBCL.

Earlier studies extensively reported that DLBCL relies on constitutive PI3K signaling to block apoptosis [6-8], indicating the important and translational significance of PI3K/AKT pathway in the clinical therapeutic intervention of DLBCL [9]. Among the different subunits of PI3K/AKT pathway, by reason of PIK3CA whose hot mutational spots have been established to be linked with carcinogenesis of different tumors, more attention was comparatively given on PIK3CA than other subunits of PI3K/AKT pathway. Consequently, we’ve selected PIK3CA as gene of interest. Actually, our previous study had already established that PIK3CA was found to be amplified in DLBCL with sample size of 60 cases using NanoString nCounter platform, the amplification percentage of which was 17% (10/60) [12]. Here, in our present investigation, we’ve extended the sample size of DLBCL tissue from 60 to 205 cases. Based on which we’ve evaluated the amplification of PIK3CA using FISH method, rather than NanoString nCounter approach employed in our previous study [12]. Notably, no significant association was observed between PIK3CA amplification and clinicopathological variables exception of overall prognosis [12], which was partially in agreement with our findings presented here that there was no significant association between PIK3CA amplification and clinicopathological variables, of course including overall prognosis. The discrepancy, in terms of association with prognosis, may be due to the different detection approaches and clinical samples we’ve used. In the case of PIK3CA amplification on DNA level in the context of lymphoma or leukemia, the related literatures were scarce and rather limited, not to mention in DLBCL. Psyrri A and colleagues detected the genetic change of PIK3CA gene in mantel cell lymphoma using quantitative real-time PCR together with FISH method, showing that amplification of PIK3CA was frequent in both mantle cell lymphoma tissues (15/22, 68%) as well as its derived cell lines. Unfortunately, they failed to analyze the clinicopathological correlation of gain of PIK3CA copy number. Therefore, there is no comparability between ours and findings from Psyrri A et al [11]. Brown JR et al found using Affymetrix 6.0 SNP array that amplification of PIK3CA occurred on 3q26 in chronic lymphocytic leukemia (CLL) [14]. Nevertheless, they’ve also failed to provide clinicopathological significance of gain of PIK3CA on DNA level in CLL. In this sense, our study was the first time to exhibit the genetic change of PIK3CA subunit on DNA level using FISH technique in DLBCL with larger sample size compared to our own previous study [12]. Furthermore, our present study totally differs from our previous study [12] in that different clinical samples used in addition to different detection method we’ve adopted. However what’s consistent between our two different studies is that, no remarkable association was observed between PIK3CA amplification and clinicopathological variables exception of overall prognosis. In the current study, it should be noted that there was the trend toward the difference of prognosis, despite no significant difference was found. DLBCL patients with gain of PIK3CA gene tend to be shorter survival than those without PIK3CA amplification. Clearly, additional studies with more cases and extended follow-up time are required to further substantiate our findings presented here. As for why PIK3CA was amplified in DLBCL remains to be further studied.

In consideration that there has been paucity of data regarding the clinicopathological significance of the PTEN loss in DLBCL; and that PTEN was the well-established inhibitor of PI3K/AKT signaling pathway [8], we’ve extended the detection of genetic change from PIK3CA to PTEN on DNA level using FISH approach. Our results obtained showed that no remarkable association was observed between PTEN loss on DNA level and clinicopathological variables available to us including overall prognosis, either. Prior numerous studies extensively reported that PTEN deficiency in cancer has been linked with advanced disease, chemotherapy resistance and poor survival [15, 16]. Nevertheless, no significant association was found between loss of PTEN versus overall prognosis in the current study. our finding was fundamentally in line with the study by Liu YY and associates showing that PTEN protein loss was not associated with patient’s clinical outcome [17], indicating that PTEN may play little prognostic role for patients with DLBCL. In terms of detection level, The vast majority of reports available related to the loss of PTEN in DLBCL, in which PTEN deficiency was commonly determined using immunohistochemistry [13, 17, 18] or western-blot [8] on protein level, which was distinctively different from our current study where PTEN deficiency was evaluated using FISH method on DNA level. Therefore, the difference in detection method could account for the discrepancy of prognostic analysis between the current study and those reporting that PTEN deficiency was markedly associated with inferior prognosis in DLBCL [15]. Given that PTEN was the negative regulator of PI3K/AKT pathway, we’ve also analyzed the correlation between loss of PTEN and PIK3CA amplification. It was observed that there was inversed but not significant correlation, which was in agreement with Pfeifer M et al’s report [8] reporting that in GCB DLBCL, the PTEN status was inversely correlated with activation of the PI3K/ AKT pathway in both DLBCL cell lines and primary patient samples. Moreover, based on the evidence of ours and those from previous other studies [8], it stands to reason that it is loss of PTEN on DNA level that may lead to the amplification of PIK3CA gene in DLBCL. As to the regulatory mechanism of PTEN over PIK3CA, it remains to be further investigated. It should be noted that evaluating PTEN loss by IHC or western-blotting as a screen method for PTEN deficiency should be carried out with caution in DLBCL. Loss of PTEN expression by IHC or western-blotting does not equate to PTEN DNA deficiency, as indicated by FISH examination. Nonetheless, if there is PTEN DNA deficiency throughout by FISH detection, one can confidently assume that there will be PTEN loss on protein level. In addition, evaluating for PTEN loss by IHC as a screen for PTEN deletion won’t provide information on the potential chromosomal aberrations that may be detected using FISH technique. Moreover, IHC result of PTEN loss may also be greatly variable with different batches of primary antibodies to PTEN antigen those previous studies employed.

Our analysis based on a larger sample size, making it unlikely that we simply missed an effect due to chance. Despite our study firstly established evidence regarding PTEN deficiency and gain of PIK3CA using FISH method on DNA level in larger sample size, there were still some limitations that deserve to be noted and stressed. A possible explanation for our null association between clinicopathological variables and genetic change of PIK3CA and PTEN is that the cut-off value we’ve set when diagnosing the genetic alterations of PIK3CA and PTEN. Given that there has been factually no agreed standard for evaluation of genetic alterations of PIK3CA and PTEN, therefore, it could be somewhat discretionary to setup the criteria or cut-off value in defining the normal and abnormal genetic change of PIK3CA and PTEN, which may lead to the potential discordance of final findings. In addition, there may be bias in the processing of clinical samples when performing FISH experiment, thereby leading to the potential artifact exists in our scoring of fluorescent signals. Thirdly, given the sensitivity of FISH testing may limit the application of FISH analysis; IHC detection could be therefore complimentary to FISH analysis [19]. Lastly, here we’re not proposing that PIK3CA amplification and PTEN loss are causality of DLBCL but just exhibit the genetic changes of both PIK3CA and PTEN were events in the pathogenesis of DLBCL. Consequently, interpretation of our results should be approached with caution that warrants further confirmation in different cohorts.

Taken together, our study showed the status of amplification of PIK3CA and loss of PTEN in DLBCL on DNA level, observing that no significant association was found between genetic changes of PIK3CA and PTEN versus clinicopathological variables available to us, including overall prognosis, suggesting that amplification of PIK3CA and loss of PTEN was an event in the pathogenesis of DLBCL.

## MATERIALS AND METHODS

### Clinical samples

The study was approved by the Medical Ethic Committee of the First Affiliated Hospital of Xinjiang Medical University. Formalin-fixed and paraffin-embedded clinical samples of DLBCL, totaling 205, were enrolled in the department of pathology. The corresponding clinicopathological information was retrieved in Hospital Information System (HIS) including age, gender, size, location, subtype, international prognosis index (IPI), performance status (PS), B-symptom, Clinical stage, Extra nodal site, concentration of lactate dehydrogenase (LDH), therapeutic effects, treatment and overall prognosis. It should be noted that not all the case enrolled whose corresponding clinicopathological information were available. Among 205 cases, there was 1 case whose age was unavailable; 15 cases whose size information was unavailable; 16 cases whose IPI was unavailable; 4 cases whose PS was unavailable; 4 cases whose B-system was unavailable; 14 cases whose clinical stage and Exln2 were unavailable; 16 cases whose LDH level was unavailable; 93 cases whose therapeutic effect was unavailable; 26 cases whose treatment was unavailable; 60 cases whose overall prognosis was unavailable. Diagnoses were histopathologically confirmed and reviewed by two separate clinical pathologists (Zhiping Ma and Mingfu Ma) on the basis of the 2008 World Health Organization (WHO) Classification. Written informed consent was obtained from each participant involved prior to undergoing biopsy.

### Fluorescent in-situ hybridization (FISH)

Fluorescence in situ hybridization (FISH) were performed to detect the genetic alterations of PIK3CA and PTEN using dual color probe containing a centromeric probe for chromosome 3 (CEN3, spectrum green) and PIK3CA probe located at 3q26 (PIK3CA, spectrum red) (ZytoLight SPEC PIK3CA/CEN 3 Dual Color Probe, Catalogue number: Z-2140-200, ZytoVision, German) or dual color probe containing a centromere probe for chromosome 10 (CEN10, spectrum green) and PTEN probe at 10q23 (PTEN, spectrum red) (Zytolight, SPEC PTEN/CEN 10 Dual Color Probe, Catalogue number: Z-2078-200, ZytoVision, German). Briefly, The DLBCL tissue microarray was de-paraffinized, air-dried and dehydrated in gradient ethnol, followed by denaturation for 5 min at 74 in 70% formamid 2×SSC solution. Hybridization was performed overnight at 37°C in a humidified chamber. Slides were subsequently washed and counterstained with 0.2μmol/L 4′-6-diamidino-2-phenylindole (DAPI) in antifade solution. Stained slides were manually interpreted with fluorescence microscope (Olympus, Japan), and the predominant FISH signal numbers were recorded in each tissue spot. For each of the cases, a minimum of 100 nonoverlapping cancer cells were evaluated for each case, totaling 100–200 cells per case. Only if ≥90% of cells demonstrated sufficient signal was the slide considered to be qualified for counting. PIK3CA amplification was considered to be present if ≥10% of nuclei [20] contained multiple PIK3CA signals and the PIK3CA /CEP3 ratio was >1 [21]. Likewise, PTEN deficiency was defined as ≥10% of nuclei contained multiple PTEN signals and the PTEN /CEP10 ratio was<1, whereas the ratio was 1 was considered the wild-type [21].

### Statistical analysis

Survival analysis was performed using Kaplan–Meier survival curve with Log-Rank test. The Fisher’s exact or χ^2^ test was used for statistical analysis of association between genetic alterations versus clinicopathological variables. The statistical analysis was performed using SPSS 17.0 software package version (Chicago, IL, USA). P value was less than 0.05 was considered to be statistically significant compared to control.
